# Occurrence and Variety of β-Lactamase Genes among *Aeromonas* spp. Isolated from Urban Wastewater Treatment Plant

**DOI:** 10.3389/fmicb.2017.00863

**Published:** 2017-05-16

**Authors:** Marta Piotrowska, Dominika Przygodzińska, Klaudia Matyjewicz, Magdalena Popowska

**Affiliations:** Department of Applied Microbiology, Institute of Microbiology, Faculty of Biology, University of WarsawWarsaw, Poland

**Keywords:** *Aeromonas*, β-lactamases, plasmid, integron, antibiotic resistance gene, horizontal gene transfer, wastewater treatment plant

## Abstract

Members of the genus *Aeromonas* that commonly occur in various aquatic ecosystems are taken into account as vectors spreading antibiotic resistance genes (ARGs) in the environment. In our study strains of *Aeromonas* spp. (*n* = 104) not susceptible to ampicillin were isolated from municipal sewage of different levels of purification – raw sewage, activated sludge and treated wastewater. The crucial step of the study was the identification of β-lactamase resistance genes. The identified genes encode β-lactamases from 14 families – *bla*_TEM_, *bla*_OXA_, *bla*_SHV_, *bla*_CTX-M_, *bla*_MOX_, *bla*_ACC_, *bla*_FOX_, *bla*_GES_, *bla*_PER_, *bla*_V EB_, *bla*_KPC_, *cphA, imiH*, and *cepH*. There were no significant differences in number of identified ARGs between isolation points. *Bla*_OXA_, *bla*_FOX_ variants and, characteristic for *Aeromonas* genus, metallo-β-lactamase *cphA*-related genes were the most commonly identified types of β-lactam resistance determinants. Moreover, we found four extended-spectrum β-lactamases (*bla*_SHV -11_, *bla*_CTX-M-27_, *bla*_CTX-M-98_, and *bla*_PER-4_) – and seven AmpC (*bla*_ACC_, *bla*_FOX-2-like_, *bla*_FOX-3_, *bla*_FOX-4-like_, *bla*_FOX-9_, *bla*_FOX-10-like_, and *bla*_FOX-13-like_) types and variants of genes that had never been found among *Aeromonas* spp. before. Five of the β-lactamases families (*bla*_TEM_, *bla*_OXA_, *bla*_FOX_, *bla*_V EB_, and *cphA*) were identified in all three isolation sites, which supports the hypothesis that wastewater treatment plants (WWTPs) are hot spots of ARGs dissemination. The obtained ARGs sequences share high identity with previously described β-lactamases, but new variants of those genes have to be considered as well. Characterization of antibiotic susceptibility was performed using disk the diffusion method with 12 different antibiotics according to CLSI guidelines. Over 60% of the strains are unsusceptible to cefepime and chloramphenicol and the majority of the strains have a multidrug resistance phenotype (68%). Finally, analysis of plasmid profiles among the resistant strains showed that 62% of the isolates from all three points of the WWTP carry plasmids of different sizes. Among some of the isolated plasmids *bla*_FOX-4-like_ and *bla*_GES_ genes have been found. To sum up, the results strongly suggest that *Aeromonas* spp. can be considered as agents of antibiotic resistance dissemination from wastewater to the natural environment.

## Introduction

The World Health Organization (WHO) identified the development of antibiotic resistance as one of the major global threats to the society and recommended intensive monitoring for the identification and surveillance of critical hot spots, aimed at reducing resistance dissemination (World Health Organization [WHO], 2013). The results of many studies show that wastewater treatment plants (WWTPs) are one of the key reservoirs of antibiotic resistance (Bouki et al., 2013). Therefore, WWTPs are now considered as one of the main hot spots of the potential emergence and spread of antibiotic resistance bacteria (ARB) and antibiotic resistance genes (ARGs) in the environment (Rizzo et al., 2013). ARB and ARGs are massively discharged into the municipal sewage system with wastewater of different origins (Kümmerer, 2009). As a reason many ARB species have been already found in WWTPs and among the most common are bacteria from the genus *Aeromonas*. Recent publications place *Aeromonas* spp. among dominant genera in wastewater communities (Chen C.-Y. et al., 2016; Hu et al., 2016). *Aeromonas* spp. are ubiquitous especially in all kind of aquatic environments, such as lakes, rivers, sea water, estuaries, pristine water, aquacultures, drinking water, or wastewater (Piotrowska and Popowska, 2014). Members of this genus are opportunistic food and waterborne pathogens of humans and animals, especially fish. From a public health point of view *Aeromonas* spp. infections are not very important, but these bacteria are taken into consideration as important vectors of ARGs in the environment (Berendonk et al., 2015).

Nowadays, there is a growing number of literature data on the dissemination of ARGs among *Aeromonas* spp. (Piotrowska and Popowska, 2014). However, the research on WWTP samples is still insufficient (Zhang et al., 2015; Varela et al., 2016). The ARGs that have been found in wastewater encode resistance to quinolones, β-lactams, aminoglycosides and tetracyclines, with the first two groups being predominant. Current research pays more attention to searching for quinolone and β-lactam resistance genes, which are potentially plasmid-mediated (Marti et al., 2014; Varela et al., 2016). However, all these studies concentrate on the most prevalent variants of these genes. In the case of β-lactam resistance genes the variety of types and variants of genes is very wide, which prompts researchers to narrow the spectrum of research and focus on a very small group of β-lactamases. So far in wastewater-derived *Aeromonas* spp. *bla*_TEM_, *bla*_OXA_, *bla*_CTX-M_, *bla*_SHV_, *bla*_KPC_, *bla*_PSE1/CARB1_, and *cphA* genes have been found (Figueira et al., 2011; Igbinosa and Okoh, 2012; Picão et al., 2013). However, the occurrence of different types of β-lactamases has been shown in studies with isolates from different environments, such as aquacultures, rivers, lakes, or sea (Henriques et al., 2006; Girlich et al., 2010, 2011). Among these strains, also the extended-spectrum β-lactamase AmpC and extended-spectrum β-lactamases (ESBL) genes *bla_FOX_, bla*_V EB_, *bla*_PER_, *bla*_GES_ have been found in addition to those listed above. In this case searching for a wider range of β-lactamase genes in wastewater seems to be justified and informative regarding the flow of these genes between different water environments.

In this study we focus on the variety of β-lactam resistance genes, which occurs among *Aeromonas* spp. isolated from wastewater of different purification levels: raw sewage, liquid phase of activated sludge and treated wastewater. The sampling points have been chosen in such a way to show the flow of β-lactam resistance genes through the treatment process. Such a diversity of β-lactamase types and variants has never been studied before in a particular wastewater environment.

## Materials and Methods

### Characteristics of Study Sites and Sample Collection

Samples of raw sewage, activated sludge and effluent were collected from the urban wastewater treatment plant (UWTP) located in Warsaw, Poland (52.351° N, 20.959° E). The UWTP is located in the north-east of Warsaw, near the Vistula river where the final effluent is discharged. Studied UWTP is a secondary treatment facility – CAS WWTP (Conventional Activated Sludge) – with average daily throughput equal to 435,300 m^3^/d, at a load of 2,400,000 PE (People Equivalents). This UWTP collects domestic, urban and hospital sewage from Warsaw and suburban area.

The strains were isolated from four time periods (September 2011, October 2011, April 2014, and June 2014) with the final pool comprising four samples of each type: raw sewage (influent), activated sludge and treated effluent (effluent). The samples were collected in 5 L sterile glass bottles, transported refrigerated to the laboratory and subjected to biological analyses within 6 h.

### Bacterial Count and Identification

A 100 mL volume of each sample was filtered through cellulose nitrate membranes (pore size 0.45 μm, Merck Millipore, Germany) and the filters were rinsed with saline. The total number of each: heterotrophs (R2Agar medium), ceftazidime resistant bacteria (R2Agar supplemented with ceftazidime) and bacteria belonging to *Aeromonas* spp. (Ryan Aeromonas Medium Base) were quantified using dilution plating procedure. Diluted (10–1000 times) and undiluted 0.1 mL aliquots were plated on R2 Agar complete medium (Graso Biotech, Poland), supplemented or not supplemented with ceftazidime or meropenem to a final concentration of 16 μg/mL, or Ryan Aeromonas Medium Base (Oxoid, England), supplemented with Ampicillin Selective Supplement (Oxoid, England). The concentrations of antibiotics were selected according to CLSI guidelines (Clinical and Laboratory Standards Institute [CLSI], 2010). R2A Agar is dedicated to the recovery and isolation of aerobic and facultative anaerobic heterotrophic bacteria. Ryan is a selective diagnostic medium for the isolation of *Aeromonas hydrophila* from clinical and environmental specimens. The plates were incubated for 24–48 h at room temperature. For each assay, plate counts were performed in triplicate with four different dilutions for each sample. Strains were stored at 4°C on agar plates supplemented with antibiotic and in LB medium with 10% glycerol at -70°C.

All isolates were identified to genus level by sequencing partial nucleotide sequence of 16S rRNA gene obtained using PCR. Amplification reactions were performed using the conditions described elsewhere (Devereux and Willis, 1995). PCR products were separated in 0.8% agarose gel by electrophoresis and purified using Clean-up Concentrator Kit (A&A Biotechnology, Poland) or Gel-out Concentrator Kit (A&A Biotechnology, Poland) according to the manufacturer’s instructions. PCR amplicons were sequenced in Genomed (Warsaw, Poland) using BigDye^®^ Terminator v3.1 from Applied Biosystems (Life Technologies) where nucleotide sequences were determined. The resulting 16S rRNA gene sequences were compared with the GenBank database using BLAST software (Altschul et al., 1990). Genus-level identifications were performed using the following criterium: a bacterium was assigned to a particular genus when identity with the genus sequences in the database was more than 95%.

### Detection of β-Lactamase Genes and Integrons

To identify β-lactamase genes, a molecular investigation by PCR amplification method was performed, mainly by Multiplex PCR using the conditions described in previous works (Perez-Perez and Hanson, 2002; Henriques et al., 2006; Dallenne et al., 2010). For this study five multiplex sets of primers and two simplex pairs of primers (Supplementary Table S1), characteristic for *Aeromonas* spp. metallo-β-lactamase chromosomal gene *cphA* and ESBL β-lactamase genes from *bla*_CTX-M8/25_ group were selected. As positive controls genomic DNA of clinical strains, obtained from the National Medicines Institute (Warsaw, Poland), carrying selected β-lactams resistance genes (Supplementary Table S2) and well-characterized foodborne *Aeromonas hydrophila* ATCC 7966 strain (Seshadri et al., 2006) were used. As a negative control *Escherichia coli* ATCC 25922 strain was used. PCR products were separated in 0.8–2% agarose gel by electrophoresis and purified using the same kits as in bacterial identification protocol. PCR products of the expected sizes were sequenced by Genomed (Warsaw, Poland). Products were compared with GenBank database, using BLAST N and BLAST X tools (Altschul et al., 1990) and with Antibiotic Resistance Database (ARDB) (Liu and Pop, 2009). Sequence analysis and assembly were performed using Clone Manager 8 (Sci-Ed Software, USA) and chromatogram viewer FinchTV (Geospiza, USA). Reference sequences have been taken from Lahey Clinic β-lactamases database^1^.

Integrase genes *intI1, intI2*, and *intI3* were identified in total DNA from isolated strains by PCR using specific primers as described previously (Henriques et al., 2006). As positive controls genomic DNA of *Aeromonas* spp. strains with confirmed presence of integrase genes was used (environmental collection of our research group). The obtained products were processed in the same way as ARGs, i.e., they were sequenced and compared with GenBank database using BLAST N.

*Bla*_FOX_ sequences were aligned using the built-in MUSCLE (default parameters), and a phylogenetic tree was built using the Neighbor joining method with default parameters and 1000 bootstrap replications with the MEGA6 software. Accession numbers for the reference genes are as follows: *bla*_FOX-1_ from *Klebsiella pneumoniae* – NG_049098.1; *bla*_FOX-2_ from *E. coli* – NG_049102.1; *bla*_FOX-3_ from *Klebsiella oxytoca* – NG_049103.1; *bla*_FOX-4_ from *E. coli* – NG_049104.1; *bla*_FOX-9_ from *K. pneumoniae* – NG_049108.1; *bla*_FOX-10_ from *K. pneumoniae* – NG_049099.1; *bla*_FOX-13_ from *Providencia rettgeri* – NG_049101.1.

### Antibiotic Susceptibility Testing and Determination of Multiple Antibiotic Resistance (MAR) Index

The susceptibility to 12 antibiotics was determined using the agar diffusion method and Clinical and Laboratory Standards Institute guidelines M45A2E (Clinical and Laboratory Standards Institute [CLSI], 2010). The antibiotics tested were ciprofloxacin (CIP, 5 μg), tetracycline (TET, 30 μg), amikacin (AK, 30 μg), gentamicin (CN, 10 μg), chloramphenicol (C, 30 μg), ceftazidime (CAZ, 30 μg), cefotaxime (CTX, 30 μg), cefepime (FEP, 30 μg), aztreonam (AZT, 30 μg), ertapenem (ERT, 10 μg), imipenem (IMP, 10 μg), and meropenem (MEM, 10 μg). Inhibition zones larger than R (resistant) and smaller than S (susceptible) were classified as intermediate resistance and excluded from the resistance percentage calculations. *E. coli* ATCC 25922 and *Pseudomonas aeruginosa* ATCC 27853 strains were included as quality controls.

The Multiple Antibiotic Resistance (MAR) index was calculated for each isolate based on the results of the disk diffusion method analysis. The MAR index for a single isolate was calculated as the number of antibiotics to which an isolate is resistant (a) divided by the total number of antibiotics against which the isolate was tested (b) (Zhang et al., 2015).

### Plasmids Isolation and Conjugation Experiments

In order to determine the location of β-lactamase resistance genes, plasmid DNA was extracted and purified using Plasmid Mini AX Gravity kit (A&A Biotechnology, Poland), according to the manufacturer’s instruction. Samples were separated by electrophoresis in 0.8% agarose gels.

### Southern Hybridization Protocol

Plasmid DNA was separated by electrophoresis in 0.8% agarose gels. Gels were then stained in ethidium bromide, and visualized using UV transilluminator. DNA was transferred to a nylon membrane (Roche Diagnostics GmbH, Germany) and hybridized with previously prepared probes. As probes, PCR-amplified and digoxigenin (DIG)-labeled resistance genes identified at the previous stages of this research were used. The process of preparing labeled probes and hybridization was performed using DIG-High Prime DNA Labeling and Detection Starter Kit I (Roche Applied Science, Germany), following the manufacturer’s procedure.

### Statistical Analyses

Statistical analyses were performed using the R 3.2.5 software (R Core Team, 2016). The prevalence of antibiotic resistance phenotypes and ARGs were compared among strains from different origin of isolations: influent, activated sludge, and effluent. In case of antibiotic resistance phenotypes generalized linear model – GLM with Bernoulli distribution was applied. Significance of the distribution of ARGs was determined using chi-square test. In both tests the approved level of significance was *p*-value < 0.05.

## Results

### Bacterial Count and Identification of *Aeromonas* Isolates

Plate counting was performed from four independent isolations. These data are shown in **Table 1**. In each replicate the total number of heterotrophic bacteria was the highest in samples from activated sludge and the lowest in the effluent from 1 × 10^7^ to 4.2 × 10^7^ and from 2.2 × 10^4^ to 5.6 × 10^4^, respectively. As the present study was aimed to detect *Aeromonas* spp. resistant to β-lactams, we applied three different media to obtain as many as possible non-repetitive isolates. The percentage of ceftazidime resistant bacteria was the highest in influent and varied between 39.8 and 0.8% and the lowest in activated sludge – from 4.9 to 1.0%; both were therefore higher than the percentage of meropenem resistant bacteria, which varied between 1.6 and 0.1% in the influent and from 0.4 to 0.001% in activated sludge. The total number of *Aeromonas* spp. isolates was assessed only twice, but each time the percentage of isolates was the highest in the effluent, ranging from 9.1 to 6.5%; in the influent it varied from 5.3 to 2.2% and in activated sludge – from 0.7 to 0.2%, being the lowest result.

**Table 1:** Total cultivable heterotrophs, *Aeromonas* spp. and ceftazidime and meropenem resistance bacterial counts (expressed in CFU/mL) and percentage of resistant isolates (% resistants) isolated from influent, activated sludge and effluent of UWTP.

Time	Sampling point	Total heterotrophs	Total ceftazidime resistant	% Resistants	Total meropenem resistant	% Resistant	Total *Aeromonas*	% *Aeromonas*
September 2011	Influent	2.40E+06	9.56E+05	39.8	3.90E+04	1.6	nd	–
	Activated sludge	1.00E+07	4.24E+05	4.2	4.47E+04	0.4	nd	–
	Effluent	2.30E+04	1.14E+03	5.0	4.90E+01	0.2	nd	–
October 2011	Influent	1.43E+06	4.80E+05	33.6	5.00E+03	0.3	nd	–
	Activated sludge	4.20E+07	8.80E+05	2.1	7.50E+04	0.2	nd	–
	Effluent	2.20E+04	1.20E+03	5.5	2.10E+02	1.0	nd	–
April 2014	Influent	1.00E+07	8.00E+04	0.8	6.00E+03	0.1	5.30E+05	5.3
	Activated sludge	1.02E+07	5.00E+05	4.9	1.00E+04	0.1	7.05E+04	0.7
	Effluent	5.00E+04	2.00E+03	4.0	5.00E+02	1.0	3.25E+03	6.5
June 2014	Influent	4.30E+06	5.60E+05	13.0	4.52E+03	0.1	9.63E+04	2.2
	Activated sludge	2.23E+07	2.13E+05	1.0	2.54E+02	0.0	4.50E+04	0.2
	Effluent	8.60E+04	2.20E+03	2.6	2.30E+01	0.0	7.80E+03	9.1

Finally, 104 non-repetitive, phenotypically and morphologically different isolates were identified as *Aeromonas* spp.: 39 from influent, 45 from activated sludge, and 20 from effluent. Species level identification was impossible to obtain, as according to literature data and our own experience the gene encoding 16S rRNA is not enough diverse, which means that among *Aeromonas* spp. this sequence is highly conserved (Janda and Abbott, 2010). In order to determine the species of identified *Aeromonas* strains, sequencing of other housekeeping genes, such as *gyrB* and *rpoD*, is necessary.

### Identification of β-Lactamases and Integron Genes

The crucial step of this study was to determine the variety of β-lactamase genes among isolated *Aeromonas* spp. strains. Finally, 13 variants of 14 types of β-lactamase genes were identified (**Table 2** and Supplementary Tables S3–S5). According to the PCR products sequencing results, identified ARGs belong to *bla*_TEM_, *bla*_OXA_, *bla*_SHV_, *bla*_CTX-M_, *bla*_MOX_, *bla*_ACC_, *bla*_FOX_, *bla*_GES_, *bla*_PER_, *bla*_V EB_, *bla*_KPC_, *cphA, imiH*, and *cepH* types. The most abundant β-lactamase-encoding genes were *bla*_OXA,_ which were found in 35.58% of strains – 36 isolates (**Table 2**). The second most prevalent β-lactamase genes were *bla*_FOX_ and *cphA*, which were identified in 29.81% (31 isolates) and 27.88% (29 isolates) of strains, respectively.

**Table 2:** Number of identified *bla, cphA, imiH, cepH* and integrase genes and their variants among isolated *Aeromonas* strains with division into three points of isolation: (a) influent – 39 resistant strains, (b) activated sludge – 45 resistant strains, and (c) effluent – 20 resistant strains.

Types of ARG	Influent	Activated sludge	Effluent	Summary
*bla*_TEM_	3	13	2	18
*bla*_OXA_	15	16	5	36
*bla*_SHV_	2	1	0	3
*bla*_SHV -11_	–	1	–	1
*bla*_SHV -12_	2	–	–	2
*bla*_CTX-M_	1	1	0	2
*bla*_CTX-M-27/98_	1			1
*bla*_CTX-M-15_	–	1		1
*bla*_ACC_	1	1	–	2
*bla*_MOX_	0	0	2	2
*bla*_MOX-10/11_	–	–	1	1
*bla*_MOX-4/8_	–	–	1	1
*bla*_FOX_	15	9	7	31
*bla*_FOX-4-like_	7	4	4	15
*bla*_FOX-10-like_	4	1	2	7
*bla*_FOX-3_	2	2	–	4
*bla*_FOX-9_	2	–	–	2
*bla*_FOX-1_	–	1	–	1
*bla*_FOX-2-like_	–	–	1	1
*bla*_FOX-13-like_	–	1	–	1
*bla*_GES_	7	9	0	16
*bla*_GES_	6	9	–	15
*bla*_GES-7_	1	–	–	1
*bla*_PER_	6	3	0	9
*bla*_PER-3_	2	1	–	3
*bla*_PER-4_	3	–	–	3
*bla*_PER-1/5_	1	2	–	3
*bla*_V EB_	3	3	3	9
*bla*_KPC_	0	2	0	2
*cphA*	4	19	6	29
*imiH*	–	4	–	4
*cepH*	–	1	–	1
*intI*	30	31	14	75
*intIII*	5	2	1	8

*Bla*_TEM_ genes were observed among 18 *Aeromonas* spp. isolates: 3 from influent, 13 from activated sludge, and 2 from effluent. The products of primers that were used in this project have 800 bp and cover the *bla*_TEM_ sequences from 13 to 812 bp (from 5 to 270 aa). Based on the databases *bla*_TEM_ genes have 861 bp and 286 aa, and variable regions have been found over the entire length of the gene. As the results of comparing our products with reference sequences, using BLAST N tool, our *bla*_TEM_ genes were 99–100% identical to different variants of *bla*_TEM_: *bla*_TEM-1_, *bla*_TEM-84_ *bla*_TEM-104_, *bla*_TEM-122,_ and *bla*_TEM-163_ (J01749, AF427130, AF516719, AY307100, and EU815939). However, using BLAST X all sequences shared 93–100% identity to *bla*_TEM-1_. The reason for these differences are changes in amino acids that are outside the PCR product sequences. For instance, between TEM-1 and TEM-84 proteins there is a single mutation at position 272 (D to N). Consequently, it is impossible to determine the variants unambiguously and they all have been classified as *bla*_TEM_.

From all identified *bla*_OXA_ genes only one gene from *Aeromonas* sp. 217, which was isolated from activated sludge, showed 99% nucleotide and amino acid identity to *bla*_OXA-47_ (NG_049751.1) and *bla*_OXA-392_ variants (NG_049683.1). The rest of the strains from influent (15 out of 39), activated sludge (16 out of 45) and effluent (5 out of 20) were 100% identical to *bla*_OXA-1_ (NG_049392.1) and other variants of these genes (NG_049613.1, GQ924769). Therefore it is also impossible to determine the variant unambiguously, based on this partial sequence.

Genes of *bla*_SHV_ β-lactamases were identified in two isolates from the influent and one isolate from activated sludge. Both strains in the influent possessed *bla* genes that showed 100% identity to *bla*_SHV -12_ variant (AJ920369). However, *bla* gene from *Aeromonas* sp. 368A isolate, which was discovered in activated sludge, belonged to a different variant and was 100% identical to *bla*_SHV -11_ (JX268754.1).

As for the *bla*_CTX-M_ genes, two strains with three different variants were found in the influent and activated sludge. *Aeromonas* sp. strain 6.41 from influent had a *bla* gene, which was 100% identical to *bla*_CTX-M-98_ (HM755448) and *bla*_CTX-M-27_ (AY156923), showing high identity to CTX-M-9 group of β-lactamases of this type. *Aeromonas* sp. strain T32 from activated sludge had a *bla* gene with 100% identity to *bla*_CTX-M-15_ (KT459668.1), which belongs to CTX-M-1 group of these β-lactamases (Bonnet, 2004).

Among the searched *ampC* genes only *bla*_MOX_, *bla*_ACC_, *cepH*, and *bla*_FOX_ types were found. In one *Aeromonas* sp. strain 104 from influent and *Aeromonas* sp. strain 368A from activated sludge *bla*_ACC_ genes were found with 100% identity to *bla*_ACC-1_ (NG_048588.1) and *bla*_ACC-4_ (NG_048594.1). Again, it was impossible to determine the variant unambiguously. Two β-lactamase genes that represent *bla*_MOX_ type were observed only in isolates from the effluent: in *Aeromonas* sp. strain AWY18 there was 100% nucleotide and amino acid identity to *bla*_MOX-4_ (NG_049317.1) and *bla*_MOX-8_ (NG_049321.1). In *Aeromonas* sp. strain 415 there was 100% identity to *bla*_MOX-11_ (NG_049313.1) and *bla*_MOX-10_ (NG_049312.1). There was no other type of *bla* gene characteristic only of the effluent. In *Aeromonas* sp. AKP12 from activated sludge several ARGs were identified, showing 95% identity to *cepH* gene (NG_047628.1).

**FIGURE 1:**
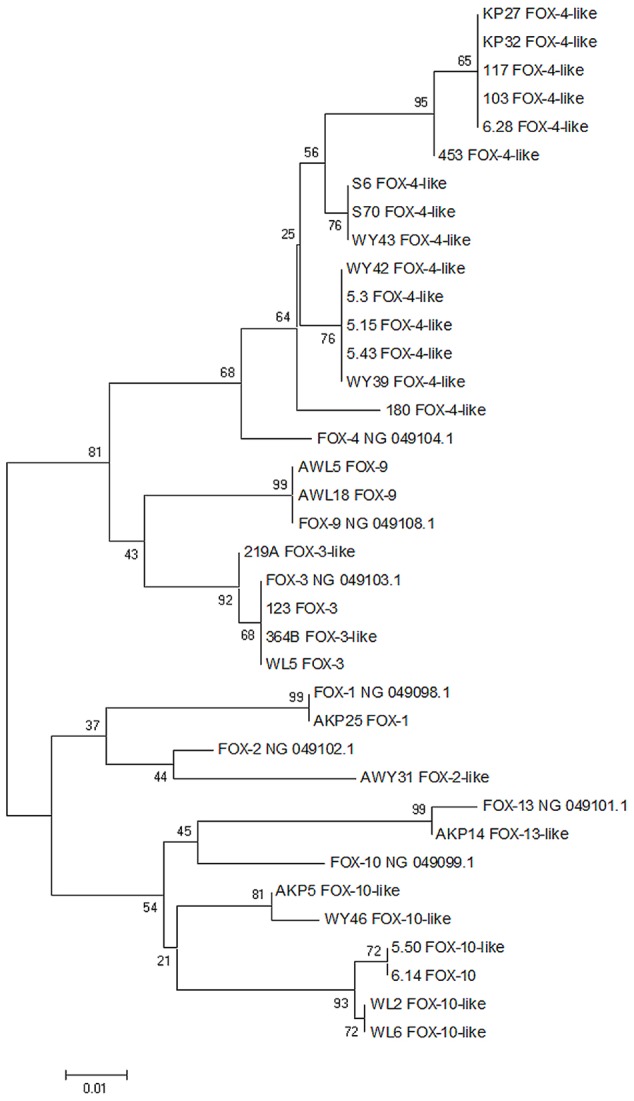
**Neighbor joining dendrogram tree based on *bla*_FOX_ gene sequences of *Aeromonas* spp. isolated from WWTP.** Seven GenBank reference *bla*_FOX_ sequences also have been attached to the analysis (sequences with NG numbers). The branch numbers refer to the percent confidence as estimated by bootstrap analysis with 1000 replications.

The most variable and numerous group of identified *ampC* genes belongs to *bla*_FOX_ β-lactamases. Genes from this type observed in this study were classified into seven different variants: *bla*_FOX-1_, *bla*_FOX-2_, *bla*_FOX-3_, *bla*_FOX-4_, *bla*_FOX-9_, *bla*_FOX-10_, and *bla*_FOX-13_. Two most prevalent variants that have been found in all three points of isolation are *bla*_FOX-4_ and *bla*_FOX-10_ genes. In total, *bla*_FOX-4_ variant was found in 16 *Aeromonas* sp. strains. However, the obtained nucleotide sequences were not identical to the deposited reference sequence. Fifteen isolates – seven from influent, four from activated sludge, and four from effluent – showed different nucleotide identity ranging from 95 to 97% to the *bla*_FOX-4_ variant of the gene (NG_049104.1). Phylogenetic analysis resulted in five possible diverse phylogenetic branches for genes identified as this variant (**Figure 1**). In the case of *bla*_FOX-10_ identified among seven *Aeromonas* sp., all of them showed 94–95% identity to *bla*_FOX-10_ variant from the database (NG_049099.1). Moreover, phylogenetic analyses divided them into two different branches. In our study only *bla*_FOX-3_ was observed in the influent and activated sludge – in each place two isolates carried this type of gene. All of them were 100% identical to *bla*_FOX-3_ (NG_049103.1) and on the phylogenetic tree all four strains were located within the same branch. The rest of identified *bla*_FOX_ variants were unique to the site of isolation and they were: *bla* from *Aeromonas* sp. AKP25 100% identical to *bla*_FOX-1_ (NG_049098.1), *bla* from *Aeromonas* sp. AWY31 97% identical to *bla*_FOX-2_ (NG_049102.1), two *bla* genes with 100% identity to *bla*_FOX-9_ (NG_049108.1) and *bla* from *Aeromonas* sp. AKP14 with 99% identity to *bla*_FOX-13_ (NG_049101.1). All identified *bla*_FOX_ variants have been confirmed using BLAST X alignment.

Two variants of *bla*_GES_ genes were also identified. The first variant isolated from influent was observed only in *Aeromonas* sp. 6.45 with 99% nucleotide identity to *bla*_GES-7_ (NG_049139.1) and to *bla*_GES-17_ (NG_049119.1). However, BLAST X analysis resulted in 100% identity to *bla*_GES-7_ encoding β-lactamase GES-7 (WP_032490683.1). The second variant was observed in six isolates from influent and in nine cultures from activated sludge. All genes demonstrated 100% nucleotide identity to several *bla*_GES_ variants and in this case again the variable region was outside the PCR product. As a result it was impossible to conclusively determine the variant of these genes.

Furthermore, genes that encode PER β-lactamases were observed only in the influent and activated sludge and they represented three different variants: *bla*_PER-3_, *bla*_PER-4_, and *bla*_PER-1/5_. In *Aeromonas* spp. with *bla*_PER-3_ all three identified genes showed 100% identity to this variant deposited in the database (NG_049962.1). Three isolates from the influent possessed *bla*_PER-4_ genes, however, two of them were 99% identical in nucleotide sequence with gene variant from the database (NG_049963.1) and also with protein sequence of PER-4 (WP_063864593.1). A third *Aeromonas* sp. 118 strain was 100% identical with the reference gene. The last variant was found in three isolates from the influent (one strain) and activated sludge (two strains); each was 100% identical to *bla*_PER-1_ (Z21957) and *bla*_PER-5_ (FJ627180). The results of BLAST X analysis also confirmed the identified variants.

Moreover, nine *bla*_V EB_ genes were identified: in three strains from the influent, in three from the activated sludge and in three strains from the effluent. None of the *bla*_V EB_ genes that were found in this study can be classified to a particular *bla*_V EB_ variant based on the obtained part of *bla*_V EB_ sequence because the variable region was outside the PCR product, and as a result the obtained products have 100% nucleotide identity to the numerous variants of genes deposited in the database.

Finally, in the activated sludge two strains possessed *bla*_KPC_ genes: *Aeromonas* sp. 368A and *Aeromonas* sp. 386. Both genes had 100% nucleotide and protein identity to *bla*_KPC-2_ and *bla*_KPC-3_ genes (AY034847, AF395881). There was no other type of *bla* gene that was characteristic only of activated sludge.

Chromosomally encoded metallo-β-lactamase *cphA*-related genes were identified very frequently in this study. They constituted a very diverse group of genes, which were highly identical to different *cphA* variants of genes and proteins (**Table 3**). In total in 29 *Aeromonas* sp. isolates *cphA*-related genes were recognized as follows: 4 in influent, 19 in activated sludge, and 6 in effluent. Besides *cphA*, other metallo-β-lactamases have also been found – in four strains with 97–98% identity to *imiH* (NG_050414.1). All these genes were identified among strains isolated from activated sludge.

**Table 3:** *CphA* gene variants identified in *Aeromonas* spp. isolated in this study.

	*Aeromonas* spp. strain	*cphA* variant^∗^	BLAST N^∗∗^	BLAST X^∗∗∗^
Influent	6.28	*cphA1*/*cphA*	95%, *A. salmonicida* (NG_047668.1)	99%, *A. salmonicida* (WP_059112452.1)
	7.47	*cphA*/*cphA*	96%, *A. aquariorum* (JF972621.1)	98%, *A. hydrophila* (WP_024941978.1)
	WL1	*cphA1*/*cphA*	95%, *A. salmonicida* (NG_047668.1)	99%, *A. salmonicida* (WP_058395305.1)
	WL3	*cphA1*/*cphA*	96%, *A. salmonicida* (NG_047668.1)	99%, *A. salmonicida* (WP_058395305.1)
Activated sludge	T6	*cphA*/subclass B2 metallo-β-lactamase	96%, *A. aquariorum* (JF972618.1)	99%, *A. hydrophila* (WP_050559324.1)
	S6	*cphA1*/*cphA*	94%, *A. salmonicida* (NG_047668.1)	96%, *A. australiensis* (WP_040097284.1)
	S12	*cphA*/*cphA2*	96%, *A. aquariorum* (JF972621.1)	98%, *A. hydrophila* (WP_063865208.1)
	E34	*cphA*/*cphA*	96%, *A. veronii* (JF972616.1)	97%, *A. australiensis* (WP_040097284.1)
	203	*cphA7*/*cphA7*	97%, *A. jandaei* (NG_050400.1)	93%, *A. jandaei* (WP_063865212.1)
	206	*cphA7*/*cphA*	96%, *A. jandaei* (NG_050400.1)	96%, *A. hydrophila* (WP_049047121.1)
	221	*cphA7*/*cphA*	96%, *A. jandaei* (NG_050400.1)	99%, *A. hydrophila* (WP_060390377.1)
	343A	*cphA7*/*cphA*	96%, *A. jandaei* (NG_050400.1)	99%, *A. hydrophila* (WP_060390377.1)
	357A	*cphA7*/*cphA*	96%, *A. jandaei* (NG_050400.1)	99%, *A. hydrophila* (WP_060390377.1)
	364B	*cphA7*/*cphA*	95%, *A. jandaei* (NG_050400.1)	96%, *A. hydrophila* (WP_049047121.1)
	368A	*cphA7*/*cphA*	96%, *A. jandaei* (NG_050400.1)	95%, *A. hydrophila* (WP_049047121.1)
	280	*cphA*/*cphA1*	96% *A. aquariorum* (JF972625.1)	97%, *A. hydrophila* (WP_063844282.1)
	297	*cphA*/*cphA1*	96% *A. aquariorum* (JF972625.1)	100%, *A. hydrophila* (WP_060390377.1)
	KO26	*cphA*/*cphA*	94%, *A. aquariorum* (JF972618.1)	97%, *A. salmonicida* (WP_043135519.1)
	AKO1	*cphA*/*cphA*	96%, *A. aquariorum* (JF972618.1)	97%, *A. veronii* (WP_005343384.1)
	AKO16	*cphA*-like/ *cphA*-like	100%, *Aeromonas* sp. G.I10.28 (DQ447638.1)	100%, *Aeromonas* sp. G.I10.28 (ABE01851.1)
	AKP15	*cphA*/*cphA1*	99%, *A. hydrophila* (NG_047667.1)	99%, *A. hydrophila* (WP_063844282.1)
	AKP19	*cphA*-type/*cphA*	96%, *A. dhakensis* (AB765398.1)	99%, *A. hydrophila* (WP_043161524.1)
	AKP23	*cphA2*/*cphA*	97%, *A. hydrophila* (NG_050396.1)	99%, *A. hydrophila* (WP_043161524.1)
Effluent	426	*cphA1*/*cphA*	97%, *A. hydrophila* (NG_047671.1)	100%, *A. hydrophila* (WP_024945765.1)
	481	*cphA1*/*cphA*	96%, *A. hydrophila* (NG_047671.1)	100%, *A. hydrophila* (WP_024945765.1)
	483	*cphA1*/*cphA*	97%, *A. hydrophila* (NG_047671.1)	100%, *A. hydrophila* (WP_024945765.1)
	WY39	*cphA*/*cphA*	94%, *A. aquariorum* (JF972621.1)	96%, *A. allosaccharophila* (WP_042063527.1)
	WY47	*cphA1*/*cphA*	96%, *A. veronii* (NG_047669.1)	97%, *A. australiensis* (WP_040097284.1)
	AWY14	*cphA*/*cphA*	97%, *A. veronii* (JQ814285.1)	98%, *A. australiensis* (WP_040097284.1)

*^∗^Gene variant based on BLAST N/BLAST X; ^∗∗^Identity, bacterial species and accession number of the most identical alignments using BLAST N; ^∗∗∗^Identity, bacterial species and accession number of the most identical alignments using BLAST X.*

The last group of genes whose presence was determined were integrase genes – *intI1* and *intI3* (**Table 2**). Type I integrase genes were observed in 72% of all isolates (75 out of 104): in 30 strains from the influent, in 31 strains from the activated sludge and in 14 strains from the effluent. Moreover, *intI3* genes were identified in 8% of isolates (8 out of 104): in 5, 2, and 1 strain, respectively. In none of the isolated *Aeromonas* spp. strains *intI2* integrase gene was confirmed.

### Antibiotic Susceptibility Phenotypes

Among all 104 *Aeromonas* spp. isolates a high percentage of bacteria were unsusceptible to cefepime (77%) and chloramphenicol (68%) (**Table 4**). The lowest percentage of bacteria were resistant to ciprofloxacin (6%), ertapenem (8%), imipenem (10%), cefotaxime (10%), meropenem (12%), and aztreonam (20%). All identified strains were unsusceptible to amikacin. Among the majority of the isolates (68%) MDR (multidrug resistance) phenotypes were observed, which means that every MDR strain was resistant to at least three different antibiotics from three different groups. Moreover, the MAR index calculations also confirmed the high percentage of multiresistant strains. The MAR index values ranged from 0.08 (resistance to one antibiotic) to 0.75 (resistance to nine antibiotics), with the most prevalent variants of 0.33 (resistance to four antibiotics) and 0.42 (resistance to five antibiotics) – each value was represented by 19% of *Aeromonas* strains (Supplementary Table S6). The analysis between three points of isolation shows that among all isolates there were fewer cefepime resistant strains in the activated sludge and effluent than in the influent (*p*-value < 0.05). Also the number of meropenem resistant strains was lower in activated sludge than in the influent (*p*-value < 0.05).

**Table 4:** Percentage of phenotypically unsusceptible strains of *Aeromonas* spp. among ceftazidime, meropenem, and ampicillin resistant *Aeromonas* spp. isolated from influent, activated sludge, and effluent of UWTP.

Sampling point	CTX	CAZ	FEP	ATM	ERT	IMP	MEM	CIP	TE	C	CN	AK	MDR > 3
Influent	5%	56%	95%	13%	3%	3%	3%	3%	21%	67%	38%	100%	74%
Activated sludge	13%	44%	71%	27%	11%	18%	22%	11%	29%	69%	36%	100%	69%
Effluent	10%	45%	55%	20%	10%	5%	5%	0%	15%	70%	30%	100%	60%
**Summary**	**10%**	**49%**	**77%**	**20%**	**8%**	**10%**	**12%**	**6%**	**23%**	**68%**	**36%**	**100%**	**68%**

*CTX, cefotaxime; CAZ, ceftazidime; FEP, cefepime; AZT, aztreonam; ERT, ertapenem; IMP, imipenem; MEM, meropenem; CIP, ciprofloxacin; TET, tetracycline; C, chloramphenicol; CN, gentamicin; AK, amikacin; MDR, multidrug resistance strains.*

### Plasmid DNA Analysis and Determination of *bla* Genes Localization

Plasmid DNA was isolated from 62% of all *Aeromonas* spp. (64 out of 104): from 28 isolates from the influent, 25 isolates from the activated sludge and 11 isolates from the effluent. Conventional gel electrophoresis revealed that all isolates from all three places of isolation contained at last one plasmid, but most of them were considered to have more than one extrachromosomal replicon. There number of different plasmid profiles identified among the influent, activated sludge and effluent samples was 26, 19, and 11, respectively, which means that most of the strains have unique plasmid profiles. In the influent there were two profiles that were found in two different strains: the first profile in *Aeromonas* sp. 111 and *Aeromonas* sp. 115 and the second in *Aeromonas* sp. WL2 and *Aeromonas* sp. WL3. In the activated sludge two profiles were observed in three different strains each: the first profile in *Aeromonas* sp. S6, *Aeromonas* sp. S70, and *Aeromonas* sp. S50 and the second in *Aeromonas* sp. 343A, *Aeromonas* sp. 297, and *Aeromonas* sp. 357A.

According to the results of Southern blot hybridization, most of the identified *bla* genes were located on chromosomes. However, based on the same method several *bla*_GES_ and *bla*_FOX_ genes had an extrachromosomal localization. In the case of *bla*_GES_ genes, four *bla*_GES_ genes identified among influent strains – *Aeromonas* sp. 5.4, *Aeromonas* sp. 6.45, *Aeromonas* sp. 111, and *Aeromonas* sp. 115 – and one *bla*_GES-7_ variant from *Aeromonas* sp. 280 from activated sludge were located on plasmids (Supplementary Tables S3, S4). Only one *bla*_FOX-4-like_ gene from *Aeromonas* sp. S6 from activated sludge was mapped to a plasmid.

## Discussion

According to the literature data, different types of ARGs have repeatedly been identified among *Aeromonas* spp. (Piotrowska and Popowska, 2014). The most frequently found are the ARGs determining resistance to quinolones and β-lactams. We based this study on the hypothesis that bacteria from the genus *Aeromonas* are an important reservoir of acquired β-lactamase genes (Maravić et al., 2013). We chose WWTP as a study object because of the poor knowledge about the prevalence of β-lactamases in this environment and in the context of the hot spot hypothesis that has already been mentioned.

The main aim of this study was the determination of β-lactamase genes diversity in isolated *Aeromonas* spp. strains. In the literature data three principal classes of β-lactamases are observed in *Aeromonas* species, according to Ambler classification: class B metallo-β-lactamase (MBL), class C cephalosporinase, and class D penicillinase (Janda and Abbott, 2010). Recent studies discovered a growing number of class A ESBLs among *Aeromonas* spp. strains as well (Lu et al., 2010; Girlich et al., 2011). In our study we also found numerous genes from classes A, C, B and D, with the most diverse and frequent class A.

Extended-spectrum β-lactamases confer resistance to all penicillins, narrow- and extended-spectrum cephalosporins and monobactams. Carbapenemases from KPC type also belong to this group. Bush and Fisher (2011) reported that almost 600 β-lactamases has been assigned to this class so far. In our study seven types of *bla* genes from class A have been found: *bla*_TEM_, *bla*_SHV_, *bla*_CTX-M_, *bla*_GES_, *bla*_PER_, *bla*_V EB_, and *bla*_KPC_. Some of the identified variants of these genes have been observed for the first time in *Aeromonas* spp. isolates from WWTP or even among *Aeromonas* genus in general. To our knowledge *bla*_SHV -11_, *bla*_CTX-M-27_, *bla*_CTX-M-98_, and *bla*_PER_ had never been observed in *Aeromonas* spp. before but have been identified among different genera. B*la*_SHV -11_ have been found in many clinical and environmental *K. pneumoniae* strains (Alibi et al., 2015; Davies et al., 2016; Shahraki-Zahedani et al., 2016), *bla*_CTX-M-27_ in many *Enterobacteriaceae* strains (Matsumura et al., 2016; Rodrigues et al., 2016), *bla*_CTX-M-98_ in *E. coli* strains from China (Liu et al., 2015) and *bla*_PER-4_ in *Proteus vulgaris* clinical strain (NG_049963). The rest of the identified ESBL *bla* genes have been observed in *Aeromonas* spp. before and these are: *bla*_TEM_, *bla*_SHV -12_, *bla*_CTX-M-15_, *bla*_PER-3_, *bla*_GES_, and *bla*_V EB_. However, only *bla*_CTX-M-15_ had been observed previously in *Aeromonas* spp. from wastewater (Amos et al., 2014). What is worth emphasizing is that *bla*_KPC_ gene, which encodes clinically emerging carbapenemase, has also been identified among strains from activated sludge. The KPC (*K. pneumoniae* carbapenemase) enzyme has been reported to spread worldwide and among several bacterial species, such as *Enterobacteriaceae* strains, *Pseudomonas aeruginosa* and *Acinetobacter baumannii* (Bush and Fisher, 2011). Literature data describes *bla*_KPC-2_ genes among *Aeromonas* sp. isolates from a hospital effluent as well (Picão et al., 2013).

Class C β-lactamases (AmpC) are located both on chromosomes and plasmids and are represented by many types that have been found worldwide in numerous sources (Jacoby, 2009). Like the chromosomal AmpC β-lactamases, plasmid-mediated enzymes confer resistance to a broad spectrum of β-lactams, including penicillins, oxyimino-β-cephalosporins, cephamycins, and aztreonam. In this study, four types of β-lactamases encoding genes from this class have been identified: *bla*_MOX_, *bla*_ACC_, *bla*_FOX_, and *cepH*. The most prevalent and diverse was *bla*_FOX_ group with seven different variants identified. Nevertheless, only three variants of these genes had 100% identical sequences to *bla* genes deposited in the NCBI database: *bla*_FOX-1_, *bla*_FOX-3_, and *bla*_FOX-9_. The rest of the variants demonstrated lower sequence identity and in this case should be proposed as *bla*_FOX-2-like_, *bla*_FOX-4-like_, *bla*_FOX-10-like_, and *bla*_FOX-13-like_. All selected reference *bla*_FOX_ genes originate from clinical pathogens and on phylogenetic trees they are located on different but closely related branches with *bla*_FOX-like_ genes that were isolated in this study. The wide range of possible new variants of *bla*_FOX-like_ genes among environmental strains seems to be unexplained, although it was previously observed among water samples of *Aeromonas* spp. (Voolaid et al., 2013). According to our knowledge, all identified variants have never been observed among *Aeromonas* spp. wastewater isolates before and, besides *bla*_FOX-1_, neither among *Aeromonas* genus.

Among the identified *ampC* genes also *bla*_MOX_ and *bla*_ACC_ genes were found. *Bla*_MOX_ variants encoding β-lactamases are derived from *K. pneumoniae bla*_MOX-1_ and most of the variants – from *bla*_MOX-3_ to *bla*_MOX-12_ have been found among environmental *Aeromonas* spp. so far (GenBank numbers: NG_049316.1, NG_049317.1, NG_049318.1, NG_049319.1, NG_049320.1, NG_049321.1, NG_049312.1, NG_049313.1, NG_049314.1). In this study *bla*_MOX-4/8_ and *bla*_MOX-10/11_ have been identified and according to our knowledge this is the first observation of these variants in wastewater isolates. Furthermore, the identified *bla*_ACC_ genes have also been found for the first time in *Aeromonas* spp. Previously, *bla*_ACC-1_ genes were described to reside within plasmids isolated from *K. pneumoniae* and *Salmonella enterica* subsp. *enterica* (Hasman, 2005; Markovska et al., 2012).

Only one type of *bla* genes from class D was found in this study – *bla*_OXA_. Genes from *bla*_OXA_ type are widely spread in many environments, also among *Aeromonas* spp., and their name derived from their oxacillin-hydrolyzing abilities. They have been found in natural waters (Henriques et al., 2006; Picão et al., 2008), aquacultures (Jacobs and Chenia, 2007), fishes (Verner-Jeffreys et al., 2009) and wastewater as well (Moura et al., 2007; Varela et al., 2016). In our study we also found many *bla*_OXA_ genes, which was an expected result.

Class B MBLs, unique carbapenemases which require zinc ion at the active site, are widely distributed in clinical and environmental species. Among *Aeromonas* spp. few MBL β-lactamases have been found: AsbM1, IMP-19, VIM, ImiS, ImiH, and most prevalent CphA (Janda and Abbott, 2010). CphA enzymes have a very specific substrate range with high hydrolytic activity against penems and carbapenems (Segatore et al., 1993). In this study *cphA*-related genes were commonly identified among isolated *Aeromonas* spp. and they were the most divergent group of genes. All recognized *cphA* genes showed high level of identity ranging from 94 to 99% to deposited variants. Wu et al. (2012) have found 51 *cphA*-positive *Aeromonas* spp. isolates with 94% identity with *cphA* gene from database (AE036). The occurrence of *cphA*-related genes is also species-related and assignment to species level of our *Aeromonas* spp. isolates should be performed. Moreover, the rules concerning nomenclature and depositing the *cphA* genes in the databases are still unclear and unspecified, which makes the determination of new variants even more complicated.

Generally, there were no significant differences between the number of β-lactamase resistance genes among all three points of isolation. This could be explained by inefficiency of the applied technology of wastewater treatment in reducing the number of ARGs or ARB during treatment process. The dissemination of ARGs and ARB from WWTP to environment is poorly understood and depending on type of treatment and studied bacteria the results differ (Rizzo et al., 2013). However, according to the literature, three factors contribute to this constant level of ARGs: (i) the optimal nutrition conditions for *Aeromonas* spp. prevailing in the biological reactor, (ii) high constant inflow of diverse clinical and environmental *Aeromonas* spp. strains brought to the reactor along with the wastewater of different origin and (III) the accumulation of the antibiotics considered a selective pressure (Novo and Manaia, 2010; Bouki et al., 2013). Our findings also support the hypothesis that *Aeromonas* spp. play an important role as vectors in dissemination of β-lactamases into the natural environment. Besides the reduced number of *Aeromonas* spp. isolates in the final effluent, the number of ARGs was not significantly decreased.

Integrons are non-replicative genetic elements, which are able to capture and incorporate gene cassettes by site-specific recombination. Most of these elements belong to the 1st, 2nd, or 3rd class of integrons and contain *intI1, intI2*, or *intI3* integrase genes, respectively. 1st and 2nd class of integrons have been found in the WWTP with predominance of the first one (Igbinosa and Okoh, 2012). Class 1 has been described at all stages of the WWTP process among *Aeromonas* spp. isolates with different frequency. However, their existence in effluents indicates the inefficiency of the treatment process in removing them (Moura et al., 2007). A large diversity of ARGs among gene cassettes (GCs) of integrons have been found in WWTPs, including β-lactamase genes: *bla*_OXA_, *bla*_V IM-2_, *bla*_IMP_, *bla*_GES-5_, and *bla*_GES-7_ (Zhang et al., 2009; Stalder et al., 2012). In our study, as expected, many *intI1* genes have been identified in all three points of isolation. There were no significant differences between points of isolation. On the other hand, the occurrence of *intI3* genes was unexpected, which is of particular importance. Previously, class 3 integrases were observed among *Aeromonas allosaccharopila* LIM82 originated from sludge in France and it has been the only described case of *intI3* among *Aeromonas* spp. (Simo Tchuinte et al., 2016). Furthermore, most of the known class 3 integrons possess GCs encoding resistance to β-lactams, which makes this class particularly interesting for our future studies (Arakawa et al., 1995; Papagiannitsis et al., 2015).

The percentage of *Aeromonas* spp. in WWTP is low and equals up to 9.1% of all bacteria. The percentage of β-lactam unsusceptible bacteria was also low and the highest levels ranged from 39.8 to 1.6%, depending on antibiotic – ceftazidime or meropenem, respectively. However, the results of disk-diffusion phenotypical resistance tests showed a high number of MDR strains among all isolated *Aeromonas* spp. strains. The majority of the isolates were resistant to at least three different antibiotics (68%), but what is even more concerning, 25% of the strains were resistant to at least six antibiotics. This data stands in accordance with the recent study of Varela et al. (2016), in which MDR *Aeromonas* sp. strains were also predominant (92.9%). A comparison of MDR isolates between points of isolation did not yield any significant differences. This was also confirmed by means of MAR index values, which were very similar regardless of the origin of the strains. This result suggests that the studied WWTP does not reduce the level of antibiotic resistance in *Aeromonas* spp. during the treatment process, in contrast to the outcome of other studies (Zhang et al., 2015).

The great majority of studied *Aeromonas* spp. strains (77%) were unexpectedly unsusceptible to cefepime which is fourth generation cephalosporin. In comparison to the literature, the percentage of cefepime unsusceptible strains in our study was more similar to the results of Picão et al. (2013) from hospital effluent than to WWTP, which were 41.7 and 7% respectively. This is an important issue related to the literature reports which show that clinical strains of *Aeromonas* spp. are susceptible to cefepime (Wang et al., 2009; Chen P.-L. et al., 2016; Soltan Dallal et al., 2016).

The low number of carbapenem resistance among *Aeromonas* spp. isolates demonstrated in this study is similar to previous observations. In all recent works the percentage of imipenem, ertapenem, or meropenem resistance in *Aeromonas* sp. strains among WWTP isolates was at a similar level – here it reached the value of about 10% for each antibiotic. In all studies, including both clinical and environmental strains, the percentage of carbapenem resistant isolates was less than 20% (Janda and Abbott, 2010; Figueira et al., 2011; Picão et al., 2013; Varela et al., 2016).

In this project the percentage of ciprofloxacin unsusceptible *Aeromonas* spp. strains (6%) was generally lower in comparison to previous works. In a Brazilian study by Picão et al. (2013) 33.3 and 11.3% of total *Aeromonas* spp. strains isolated from hospital effluent and WWTP, respectively, were resistant to ciprofloxacin. In a Portuguese study by Figueira et al. (2011) and Varela et al. (2016) over 40 and 66% of WWTP *Aeromonas* spp. strains revealed this phenotype respectively. In Portugal, outpatient usage of quinolones was three times higher than in Poland, which could be an explanation for these differences (Ferech et al., 2006). However, the genetic background of this phenomenon should be studied in the future, especially considering the use of fluoroquinolones as one of the first choice drugs in *Aeromonas* spp. infections (Parker and Shaw, 2011).

Plasmids are ubiquitous among all prokaryotes, including those inhabiting the WWTP environment, as they play a significant role in horizontal gene transfer and interspecies dissemination of virulence and resistance determinants. In our study, as a result of determining the genomic localization of identified *bla* genes, *bla*_FOX-4-like_, *bla*_GES_ and *bla*_GES-7_ have been found within the genomes of isolated plasmids. Based on literature data, *bla*_GES-7_ gene was identified on 60 kb plasmid of *A. veronii* isolated from Seine River in France (Girlich et al., 2011). Plasmid location of *bla*_FOX_ genes has never been confirmed among *Aeromonas* spp. before. Besides that, also *bla*_OXA-1_, *bla*_OXA-10_, *bla*_CMY -2_, and *bla*_CTX-M-15_ have already been confirmed as plasmid-mediated among *Aeromonas* spp. of different environmental origin (Piotrowska and Popowska, 2015). In our study, according to our methodology, besides *bla*_GES_ and *bla*_FOX,_ the rest of identified *bla* genes were located on bacterial chromosomes.

## Conclusion

Our major findings in this study were: (i) the identification of ESBL (*bla*_SHV -11_, *bla*_CTX-M-27_, *bla*_CTX-M-98_, and *bla*_PER-4_) and AmpC (*bla*_ACC_, *bla*_FOX-2-like_, *bla*_FOX-3_, *bla*_FOX-4-like_, *bla*_FOX-9_, *bla*_FOX-10-like_, and *bla*_FOX-13-like_) variants of genes, which have never been found among *Aeromonas* spp. before; (ii) the identification of plasmid-mediated *bla*_GES_ and *bla*_FOX-4-like_ genes with the special emphasis on the second type, which have never been observed within plasmid DNA among *Aeromonas* spp. before; (iii) the lack of significant differences in number of ARGs between points of isolation and (iv) unexpectedly high number of the strains resistant to cefepime. These findings make *Aeromonas* spp. strains an important research object and in the light of these results the study of the spread of β-lactamase genes and searching for hot spots of their dissemination among *Aeromonas* spp. seems to be justified. However, our knowledge about *Aeromonas* spp. resistance in WWTPs is still not complete and requires more comprehensive and in-depth studies, as well as regular monitoring. This seems to be of particular importance especially given the increasing number of *Aeromonas* spp. infections and MDR strains that are spreading around the world (Esteve et al., 2015; Batra et al., 2016; Qamar et al., 2016).

## Author Contributions

MPi: contributed to the establishment and coordination of the collaborations, manuscript design, data collection, data analysis, and drafting and writing of the manuscript. DP and KM: contributed equally to the data collection. MPo: contributed to manuscript design, writing and editing the manuscript, coordination of research and coordination of the collaborations.

## Conflict of Interest Statement

The authors declare that the research was conducted in the absence of any commercial or financial relationships that could be construed as a potential conflict of interest.

## Footnotes

1 https://www.lahey.org
