# Supplementary material for: Occurrence and Variety of β-Lactamase Genes among Aeromonas spp. Isolated from Urban Wastewater Treatment Plant
Source: Front Microbiol. 2017 May 16;8:863. doi: 10.3389/fmicb.2017.00863 (PMC5432545; doi:10.3389/fmicb.2017.00863)
Supplement: Supplementary file 1 [file Table_1.DOCX]

**Supplementary material**

**Table 1S.** Genes that have been targeted in chosen multiplex PCR reactions (based on Perez-Perez and Hanson, 2002 and Dallenne et al., 2010)

| Multiplex | Genes |
| --- | --- |
| Multiplex I | *bla*_TEM-1_, *bla*_TEM-2_, *bla*_SHV-1_, *bla*_OXA-1_, *bla*_OXA-4_, *bla*_OXA-30_ |
| Multiplex II | *bla*_CTX-M-1_, *bla*_CTX-M-3_, *bla*_CTX-M-15_, *bla*_CTX-M-2_, *bla*_CTX-M-9_, *bla*_CTX-M-14_ |
| Multiplex III | *bla*_ACC_ variants, *bla*_FOX_ variants, *bla*_MOX_ variants, *bla*_CMY-1_, *bla*_CMY-8_ to *bla*_CMY-11_, *bla*_CMY-19_, *bla*_DHA-1_, *bla*_DHA-2_, *bla*_LAT-1_ to *bla*_LAT-4_, *bla*_BIL-1_, *bla*_CMY-2_ to *bla*_CMY-7_, *bla*_CMY-12_ to *bla*_CMY-18_, *bla*_CMY-21_ to *bla*_CMY-23_, *bla*_ACT-1_, *bla*_MIR-1_ |
| Multiplex IV | *bla*_GES-1_ to *bla*_GES-9_, *bla*_GES-11_, *bla*_PER-1_, *bla*_PER-3_, *bla*_VEB-1_ to *bla*_VEB-6_ |
| Multiplex V | *bla*_IMP_ variants, *bla*_VIM_ variants, *bla*_KPC-1_ to bla_KPC-5_ |

**Table 2S.** Clinical strains having a selected beta-lactam resistance genes which total DNA were used as a positive control strains (obtained from National Medicines Institute, Warsaw, Poland)

| Strain | Possessed beta-lactamases |
| --- | --- |
| *Pseudomonas mirabilis* 3103/03 | *bla*_CMY-12_, *bla*_OXA-1_, *bla*_TEM-2_ |
| *Klebsiella pneumoniae* 2102/06 | *bla*_DHA-1_, *bla*_OXA-1_, *bla*_SHV-1_ |
| *Escherichia coli* 3551/98 | *bla*_CTX-M-15_, *bla*_TEM-1_ |
| *Pseudomonas aeruginosa* 1956/01 | *bla*_VIM-2_ |
| *Pseudomonas aeruginosa* 2550/04 | *bla*_PER-1_, *bla*_OXA-74_, *bla*_OXA-2_ |
| *Escherichia coli* 1841/06 | *bla*_CTX-M-9_, *bla*_TEM-1_ |
| *Pseudomonas aeruginosa* 2646/06 | *bla*_IMP-7_ |
| *Escherichia coli* 3465/08 | *bla*_KPC-2_, *bla*_TEM-1_ |

**Table 3S.** Antibiotic resistance phenotype, beta-lactamases and integrase genes profile, presence of plasmids and identified plasmid localization of *bla* genes among *Aeromonas* spp. strains isolated from influent

| **Strain** | **Antibiotic resistance phenotype** | **Beta-lactamases genes** | **Integrase gene** | **Plasmid replicon** | **Plasmid localization of ARG** |
| --- | --- | --- | --- | --- | --- |
| 5.3 | FEP, TET, C, AK | *bla*_FOX-4-like_ | *intI1* | **-** |  |
| 5.4 | CAZ, FEP, TET, C, CN, AK | *bla*_GES_ | *intI1, intI3* | + | *bla*_GES_ |
| 5.15 | FEP, TET, C, AK | *bla*_FOX-4-like_ | *intI1* | - |  |
| 5.22 | CAZ, FEP, ATM, C, AK | *bla*_PER-3_ | *intI1* | - |  |
| 5.43 | C, AK | *bla*_FOX-4-like_ | *intI1* | + |  |
| 5.46 | CTX, FEP, C, CN, AK | *bla*_TEM_, *bla*_OXA_ | *intI1* | - |  |
| 5.50 | FEP, C, CN, AK | *bla*_FOX-10-like_ | *intI1* | + |  |
| 6.14 | FEP, C, AK | *bla*_OXA_, *bla*_FOX-10-like_ | *intI1* | - |  |
| 6.27 | FEP, TET, C, AK | *bla*_OXA_ | *intI1* | + |  |
| 6.28 | FEP, TET, C, AK | *bla*_OXA_, *bla*_FOX-4-like_, *cphA* | *intI1* | - |  |
| 6.41 | FEP, CIP, TET, AK | *bla*_CTX-M-27/98_ | *intI1, intI3* | + |  |
| 6.45 | CAZ, FEP, ATM, C, CN, AK | *bla*_OXA_, *bla*_GES-7_ | *intI1* | + | *bla*_GES-7_ |
| 7.47 | CAZ, FEP, ATM, TET, C, CN, AK | *cphA* | *intI1* | + |  |
| 103 | CAZ, FEP AK | *bla*_GES_, *bla*_FOX-4-like_ | *intI1* | + |  |
| 104 | CAZ, FEP, C AK | *bla*_ACC_ | *-* | - |  |
| 111 | CAZ, FEP, CN, AK | *bla*_OXA_, *bla*_GES_ | *intI1, intI3* | + | *bla*_GES_ |
| 112 | CAZ, FEP, C, CN, AK | *bla*_OXA_, *bla*_PER-1/5_ | *intI1* | + |  |
| 115 | CAZ, FEP, CN, AK | *bla*_OXA_, *bla*_GES_ | *intI1, intI3* | + | *bla*_GES_ |
| 117 | FEP, C, AK | *bla*_SHV-12_, *bla*_FOX-4-like_ | *intI1* | + |  |
| 118 | CAZ, FEP, C, CN, AK | *bla*_OXA_, *bla*_PER-4_ | *intI1* | + |  |
| 121 | CTX, CAZ, FEP, TET, CN, AK | *bla*_TEM_ | *intI1* | + |  |
| 123 | CAZ, FEP, AK | *bla*_FOX-3_ | *intI1* | + |  |
| 131 | CAZ, FEP, AK | *bla*_VEB_ | *-* | + |  |
| 137 | CAZ, FEP, C, CN, AK | *bla*_TEM_ | *intI1, intI3* | + |  |
| 143 | FEP, C, AK | *bla*_TEM_, *bla*_OXA_, *bla*_GES_ | *intI1* | + |  |
| 148 | CAZ, FEP, ATM, C, CN, AK | *bla*_PER-3_ | *intI1* | + |  |
| 180 | FEP, ERT, IMP, MEM, AK | *bla*_FOX-4-like_ | *-* | - |  |
| 185 | CAZ, FEP, C, CN, AK | *bla*_OXA_, *bla*_PER-4_ | *intI1* | + |  |
| 186 | CAZ, FEP, C, CN, AK | *bla*_OXA_, *bla*_PER-4_ | *intI1* | - |  |
| 199 | CAZ, FEP, ATM, AK | *bla*_SHV-12_ | *-* | + |  |
| WL1 | AK | *cphA* | *-* | - |  |
| WL2 | FEP, AK | *bla*_FOX-10-like_ | *-* | + |  |
| WL3 | FEP, C, AK | *cphA* | *-* | + |  |
| WL5 | CAZ, FEP, C, AK | *bla*_FOX-3_ | *intI1* | - |  |
| WL6 | FEP, AK | *bla*_FOX-10_ | *-* | + |  |
| AWL4 | CAZ, FEP, C, AK | *bla*_OXA_, *bla*_VEB_ | *intI1* | + |  |
| AWL5 | FEP, AK | *bla*_FOX-9_ | *-* | + |  |
| AWL18 | CAZ, FEP, C, CN, AK | *bla*_OXA_, *bla*_FOX-9_ | *intI1* | + |  |
| AWL24 | CAZ, FEP, C, AK | *bla*_OXA_, *bla*_VEB_ | *intI1* | + |  |

**Table 4S.** Antibiotic resistance phenotype, beta-lactamases and integrase genes profile, presence of plasmids and identified plasmid localization of *bla* genes among *Aeromonas* spp. strains isolated from activated sludge

| **Strain** | **Antibiotic resistance phenotype** | **Beta-lactamases genes** | **Integrase gene** | **Plasmid replicon** | **Plasmid localization of ARG** |
| --- | --- | --- | --- | --- | --- |
| C19 | CAZ, FEP, ATM, ERT, CIP, AK | *bla*_OXA_ | *intI1* | - |  |
| E33 | CAZ, FEP, ATM, ERT, CIP, AK | *bla*_TEM_, *bla*_OXA_ | *intI1* | - |  |
| T6 | CAZ, FEP, ATM, ERT, CIP, AK | *bla*_TEM_, *cphA* | *intI1* | + |  |
| T32 | CAZ, FEP, ATM, ERT, CIP, AK | *bla*_CTX-M-15_ | *intI1* | - |  |
| S14 | CAZ, FEP, ATM, ERT, CIP, AK | *bla*_TEM_ | *intI1* | - |  |
| S24 | CAZ, FEP, ATM, ERT, CIP, AK | *bla*_TEM_, *bla*_OXA_ | *intI1* |  |  |
| S6 | FEP, CIP, TET, C, CN, AK | *bla*_OXA_, *bla*_FOX-4-like_, *cphA* | *intI1* | + | *bla*_FOX-4-like_ |
| S12 | TET, C, AK | *cphA* | *intI1* | + |  |
| S22 | FEP, CIP, TET, C, AK | *bla*_OXA_ | *intI1* | - |  |
| S50 | FEP, TET, C, AK | *bla*_OXA_ | *intI1* | + |  |
| S70 | FEP, C, CN, AK | *bla*_OXA_, *bla*_FOX-4-like_ | *intI1* | + |  |
| E34 | FEP, C, AK | *cphA* | *intI1* | + |  |
| 203 | CTX, FEP, ATM, IMP, MEM, C, CN, AK | *bla*_GES_, *cphA* | *intI1* | + |  |
| 206 | FEP, C, AK | *cphA* | *intI1* | - |  |
| 210B | CAZ, FEP, AK | *bla*_PER-1/5_ | *-* | + |  |
| 214A | CAZ, FEP, ATM, TET, C, AK | *bla*_GES_, *imiH* | *intI1* | + |  |
| 215 | CAZ, FEP, AK | *bla*_VEB_ | *-* | + |  |
| 217 | CTX, CAZ, FEP, TET, C, AK | *bla*_OXA_, *bla*_GES_ | *intI1* | + |  |
| 219A | CAZ, FEP, ATM, C, CN, AK | *bla*_PER-1/5_, *bla*_FOX-3_ | *intI1* | + |  |
| 221 | CAZ, FEP, ATM, ERT, IMP, MEM, C, CN, AK | *bla*_GES_, *cphA* | *intI1, intI3* | - |  |
| 306 | CTX, FEP, ATM, TET, C, CN, AK | *bla*_OXA_ | *intI1* | + |  |
| 311 | CAZ, FEP, AK | *bla*_VEB_ | *-* | + |  |
| 315 | CAZ, FEP, ATM, C, AK | *bla*_PER-3_ | *intI1* | - |  |
| 343A | CAZ, FEP, ERT, IMP, MEM, C, AK | *bla*_OXA_, *bla*_GES_, *bla*_TEM_, *cphA* | *intI1* | + |  |
| 357A | CAZ, FEP, ERT, IMP, MEM, C, AK | *bla*_OXA_, *cphA* | *intI1* | + |  |
| 364B | CAZ, FEP, CN, AK | *bla*_FOX-3_, *cphA*, *bla*_TEM_ | *intI1* | + |  |
| 368A | CAZ, FEP, ATM, IMP, MEM, CIP, C, CN, AK | *bla*_TEM_, *bla*_SHV-11_, *bla*_ACC_, *bla*_KPC_, *cphA* | *intI1* | + |  |
| 386 | CAZ, FEP, ATM, MEM, C, AK | *bla*_GES_, *bla*_KPC_, *bla*_TEM_ | *intI1, intI3* | - |  |
| 280 | CAZ, IMP, MEM, C, AK | *bla*_OXA_, *bla*_GES_, *cphA*, *bla*_TEM_ | *intI1* | + | *bla*_GES_ |
| 297 | CAZ, FEP, ERT, IMP, MEM, C, AK | *bla*_OXA_, *bla*_GES_, *cphA*, *bla*_TEM_ | *intI1* | + |  |
| KO26 | AK | *cphA* | *-* | - |  |
| KP27 | AK | *bla*_FOX-4-like_ | *-* | + |  |
| AKO1 | AK | *cphA* | *-* | - |  |
| AKO6 | AK | *imiH* | *-* | - |  |
| AKO8 | MEM, AK | *imiH* | *-* | - |  |
| AKO16 | TET, C, AK | *cphA* | *-* | + |  |
| KP32 | FEP, AK | *bla*_FOX-4-like_ | *-* | + |  |
| AKP5 | FEP, TET, C, CN, AK | *bla*_TEM_, *bla*_OXA_, *bla*_FOX-10-like_, *bla*_VEB_ | *intI1* | - |  |
| AKP7 | C, AK | *imiH* | *-* | - |  |
| AKP12 | AK | *cepH* | *-* | - |  |
| AKP14 | FEP, TET, C, CN, AK | *bla*_TEM_, *bla*_OXA_, bla_FOX-13-like_ | *intI1* | + |  |
| AKP15 | AK | *cphA* | *-* | - |  |
| AKP19 | FEP, IMP, AK | *cphA* | *-* | - |  |
| AKP23 | C, AK | *cphA* | *-* | - |  |
| AKP25 | CAZ, FEP, MEM, C, CN, AK | *bla*_TEM_, *bla*_OXA_, *bla*_GES_, *bla*_FOX-1_ | *intI1* | + |  |

**Table 5S**. Antibiotic resistance phenotype, beta-lactamases and integrase genes profile, presence of plasmids and identified plasmid localization of *bla* genes among *Aeromonas* spp. strains isolated from effluent

| **Strain** | **Antibiotic resistance phenotype** | **Beta-lactamases genes** | **Integrase gene** | **Plasmid replicon** | **Plasmid localization of ARG** |
| --- | --- | --- | --- | --- | --- |
| S10B | CAZ, FEP, ATM, C, AK | *bla*_VEB_ | *intI1* | - |  |
| S16 | CAZ, FEP, ATM, C, AK | *bla*_VEB_ | *intI1* | - |  |
| 406 | CAZ, FEP, C, AK | *bla*_OXA_ | *intI1* | + |  |
| 415 | CAZ, FEP, C, AK | *bla*_MOX-10/11_ | *intI1* | - |  |
| 426 | CAZ, FEP, ATM, ERT, C, CN, AK | *bla*_OXA_, *cphA* | *intI1* | + |  |
| 453 | AK | *bla*_FOX-4-like_ | *-* | + |  |
| 458 | CTX, CAZ, FEP, C, CN, AK | *bla*_TEM_ | *intI1* | + |  |
| 481 | CAZ, FEP, ATM, ERT, IMP, MEM, C, CN, AK | *bla*_TEM_, *bla*_OXA_, *cphA* | *intI1, intI3* | + |  |
| 483 | CTX, CAZ, FEP, C, AK | *cphA* | *intI1* | - |  |
| WY38 | C, AK | *bla*_FOX-10-like_ | *-* | - |  |
| WY39 | AK | *bla*_FOX-4-like_, *cphA* | *-* | - |  |
| WY42 | AK | *bla*_FOX-4-like_ | *-* | + |  |
| WY43 | AK | *bla*_FOX-4-like_ | *-* | + |  |
| WY46 | FEP, TET, AK | *bla*_FOX-10-like_ | *intI1* | + |  |
| WY47 | TET, AK | *cphA* | *-* | + |  |
| WY50 | FEP, TET, C, AK | *bla*_OXA_ | *intI1* | + |  |
| AWY13 | CAZ, FEP, C, CN, AK | *bla*_VEB_ | *intI1* | - |  |
| AWY14 | C, AK | *cphA* | *intI1* | + |  |
| AWY18 | C, AK | *bla*_MOX-4/8_ | *intI1* | - |  |
| AWY31 | C, CN, AK | *bla*_OXA_, *bla*_FOX-2-like_ | *intI1* | - |  |

**Table 6S.** Antibiotic susceptibility to 12 selected antibiotics among *Aeromonas* spp. isolates isolated from influent (I), activated sludge (AS) and effluent (E) of UWTP

| Strain | **CTX** | **CAZ** | **FEP** | **ATM** | **ERT** | **IMP** | **MEM** | **CIP** | **TET** | **C** | **CN** | **AK** | **Sampling point** | **Number of  antibiotic resistance** | **MAR index** | **MDR** |
| --- | --- | --- | --- | --- | --- | --- | --- | --- | --- | --- | --- | --- | --- | --- | --- | --- |
| 5.3 | S | S | R | S | S | S | S | S | R | R | S | R | I | 4 | 0,33 | + |
| 5.4 | S | R | R | S | S | S | S | S | R | R | R | R | I | 6 | 0,50 | + |
| 5.15 | S | S | R | S | S | S | S | S | R | R | S | R | I | 4 | 0,33 | + |
| 5.22 | S | R | R | R | S | S | S | S | S | R | S | R | I | 5 | 0,42 | + |
| 5.43 | S | S | S | S | S | S | S | S | S | R | S | R | I | 2 | 0,17 | - |
| 5.46 | R | S | R | S | S | S | S | S | S | R | R | R | I | 5 | 0,42 | + |
| 5.50 | S | S | R | S | S | S | S | S | S | R | R | R | I | 4 | 0,33 | + |
| 6.14 | S | S | R | S | S | S | S | S | S | R | S | R | I | 3 | 0,25 | + |
| 6.27 | S | S | R | S | S | S | S | S | R | R | S | R | I | 4 | 0,33 | + |
| 6.28 | S | S | R | S | S | S | S | S | R | R | S | R | I | 4 | 0,33 | + |
| 6.41 | S | S | R | S | S | S | S | R | R | S | S | R | I | 4 | 0,33 | + |
| 6.45 | S | R | R | R | S | S | S | S | S | R | R | R | I | 6 | 0,50 | + |
| 7.47 | S | R | R | R | S | S | S | S | R | R | R | R | I | 7 | 0,58 | + |
| 103 | S | R | R | S | S | S | S | S | S | S | S | R | I | 3 | 0,25 | - |
| 104 | S | R | R | S | S | S | S | S | S | R | S | R | I | 4 | 0,33 | + |
| 111 | S | R | R | S | S | S | S | S | S | S | R | R | I | 4 | 0,33 | + |
| 112 | S | R | R | S | S | S | S | S | S | R | R | R | I | 5 | 0,42 | + |
| 115 | S | R | R | S | S | S | S | S | S | S | R | R | I | 4 | 0,33 | + |
| 117 | S | S | R | S | S | S | S | S | S | R | S | R | I | 3 | 0,25 | + |
| 118 | S | R | R | S | S | S | S | S | S | R | R | R | I | 5 | 0,42 | + |
| 121 | R | R | R | S | S | S | S | S | R | S | R | R | I | 6 | 0,50 | + |
| 123 | S | R | R | S | S | S | S | S | S | S | S | R | I | 3 | 0,25 | - |
| 131 | S | R | R | S | S | S | S | S | S | S | S | R | I | 3 | 0,25 | - |
| 137 | S | R | R | S | S | S | S | S | S | R | R | R | I | 5 | 0,42 | + |
| 143 | S | S | R | S | S | S | S | S | S | R | S | R | I | 3 | 0,25 | + |
| 148 | S | R | R | R | S | S | S | S | S | R | R | R | I | 6 | 0,50 | + |
| 180 | S | S | R | S | R | R | R | S | S | S | S | R | I | 5 | 0,42 | - |
| 185 | S | R | R | S | S | S | S | S | S | R | R | R | I | 5 | 0,42 | + |
| 186 | S | R | R | S | S | S | S | S | S | R | R | R | I | 5 | 0,42 | + |
| 199 | S | R | R | R | S | S | S | S | S | S | S | R | I | 4 | 0,33 | - |
| WL1 | S | S | S | S | S | S | S | S | S | S | S | R | I | 1 | 0,08 | - |
| WL2 | S | S | R | S | S | S | S | S | S | S | S | R | I | 2 | 0,17 | - |
| WL3 | S | S | R | S | S | S | S | S | S | R | S | R | I | 3 | 0,25 | + |
| WL5 | S | R | R | S | S | S | S | S | S | R | S | R | I | 4 | 0,33 | + |
| WL6 | S | S | R | S | S | S | S | S | S | S | S | R | I | 2 | 0,17 | - |
| AWL4 | S | R | R | S | S | S | S | S | S | R | S | R | I | 4 | 0,33 | + |
| AWL5 | S | S | R | S | S | S | S | S | S | S | S | R | I | 2 | 0,17 | - |
| AWL18 | S | R | R | S | S | S | S | S | S | R | R | R | I | 5 | 0,42 | + |
| AWL24 | S | R | R | S | S | S | S | S | S | R | S | R | I | 4 | 0,33 | + |
| E33 | R | S | R | S | S | S | S | S | S | R | R | R | AS | 5 | 0,42 | + |
| C19 | S | R | R | R | R | S | S | R | S | S | S | R | AS | 6 | 0,50 | + |
| T6 | S | R | R | R | S | S | S | S | R | R | R | R | AS | 7 | 0,58 | + |
| T32 | R | R | R | R | S | S | S | R | R | R | R | R | AS | 9 | 0,75 | + |
| S14 | S | S | S | S | S | S | S | S | R | R | R | R | AS | 4 | 0,33 | + |
| S24 | R | R | R | R | S | S | S | S | S | R | R | R | AS | 7 | 0,58 | + |
| S6 | S | S | R | S | S | S | S | R | R | R | R | R | AS | 6 | 0,50 | + |
| S12 | S | S | S | S | S | S | S | S | R | R | S | R | AS | 3 | 0,25 | + |
| S22 | S | S | R | S | S | S | S | R | R | R | S | R | AS | 5 | 0,42 | + |
| S50 | S | S | R | S | S | S | S | S | R | R | S | R | AS | 4 | 0,33 | + |
| S70 | S | S | R | S | S | S | S | S | S | R | R | R | AS | 4 | 0,33 | + |
| E34 | S | S | R | S | S | S | S | S | S | R | S | R | AS | 3 | 0,25 | + |
| 203 | R | S | R | R | S | R | R | S | S | R | R | R | AS | 8 | 0,67 | + |
| 206 | S | S | R | S | S | S | S | S | S | R | S | R | AS | 3 | 0,25 | + |
| 210B | S | R | R | S | S | S | S | S | S | S | S | R | AS | 3 | 0,25 | - |
| 214A | S | R | R | R | S | S | S | S | R | R | S | R | AS | 6 | 0,50 | + |
| 215 | S | R | R | S | S | S | S | S | S | S | S | R | AS | 3 | 0,25 | - |
| 217 | R | R | R | S | S | S | S | S | R | R | S | R | AS | 6 | 0,50 | + |
| 219A | S | R | R | R | S | S | S | S | S | R | R | R | AS | 6 | 0,50 | + |
| 221 | S | R | R | R | R | R | R | S | S | R | R | R | AS | 9 | 0,75 | + |
| 306 | R | S | R | R | S | S | S | S | R | R | R | R | AS | 7 | 0,58 | + |
| 311 | S | R | R | S | S | S | S | S | S | S | S | R | AS | 3 | 0,25 | - |
| 315 | S | R | R | R | S | S | S | S | S | R | S | R | AS | 5 | 0,42 | + |
| 343A | S | R | R | S | R | R | R | S | S | R | S | R | AS | 7 | 0,58 | + |
| 357A | S | R | R | S | R | R | R | S | S | R | S | R | AS | 7 | 0,58 | + |
| 364B | S | R | R | S | S | S | S | S | S | S | R | R | AS | 4 | 0,33 | + |
| 368A | S | R | R | R | S | R | R | R | S | R | R | R | AS | 9 | 0,75 | + |
| 386 | S | R | R | R | S | S | R | S | S | R | S | R | AS | 6 | 0,50 | + |
| 280 | S | R | S | S | S | R | R | S | S | R | S | R | AS | 5 | 0,42 | + |
| 297V | S | R | R | S | R | R | R | S | S | R | S | R | AS | 7 | 0,58 | + |
| KO26 | S | S | S | S | S | S | S | S | S | S | S | R | AS | 1 | 0,08 | - |
| KP27 | S | S | S | S | S | S | S | S | S | S | S | R | AS | 1 | 0,08 | - |
| AKO1 | S | S | S | S | S | S | S | S | S | S | S | R | AS | 1 | 0,08 | - |
| AKO6 | S | S | S | S | S | S | S | S | S | S | S | R | AS | 1 | 0,08 | - |
| AKO8 | S | S | S | S | S | S | R | S | S | S | S | R | AS | 2 | 0,17 | - |
| AKO16 | S | S | S | S | S | S | S | S | R | R | S | R | AS | 3 | 0,25 | + |
| KP32 | S | S | R | S | S | S | S | S | S | S | S | R | AS | 2 | 0,17 | - |
| AKP5 | S | S | R | S | S | S | S | S | R | R | R | R | AS | 5 | 0,42 | + |
| AKP7 | S | S | S | S | S | S | S | S | S | R | S | R | AS | 2 | 0,17 | - |
| AKP12 | S | S | S | S | S | S | S | S | S | S | S | R | AS | 1 | 0,08 | - |
| AKP14 | S | S | R | S | S | S | S | S | R | R | R | R | AS | 5 | 0,42 | + |
| AKP15 | S | S | S | S | S | S | S | S | S | S | S | R | AS | 1 | 0,08 | - |
| AKP19 | S | S | R | S | S | R | S | S | S | S | S | R | AS | 3 | 0,25 | - |
| AKP23 | S | S | S | S | S | S | S | S | S | R | S | R | AS | 2 | 0,17 | - |
| AKP25 | S | R | R | S | S | S | R | S | S | R | R | R | AS | 6 | 0,50 | + |
| S10B | S | R | R | R | S | S | S | S | S | R | S | R | E | 5 | 0,42 | + |
| S16 | S | R | R | R | S | S | S | S | S | R | S | R | E | 5 | 0,42 | + |
| 406 | S | R | R | S | S | S | S | S | S | R | R | R | E | 5 | 0,42 | + |
| 415 | S | R | R | S | S | S | S | S | S | R | S | R | E | 4 | 0,33 | + |
| 426 | S | R | R | R | R | S | S | S | S | R | R | R | E | 7 | 0,58 | + |
| 453 | S | S | S | S | S | S | S | S | S | S | S | R | E | 1 | 0,08 | - |
| 458 | R | R | R | S | S | S | S | S | S | R | R | R | E | 6 | 0,50 | + |
| 481 | S | R | R | R | R | R | R | S | S | R | R | R | E | 9 | 0,75 | + |
| 483 | R | R | R | S | S | S | S | S | S | R | S | R | E | 5 | 0,42 | + |
| WY38 | S | S | S | S | S | S | S | S | S | R | S | R | E | 2 | 0,17 | - |
| WY39 | S | S | S | S | S | S | S | S | S | S | S | R | E | 1 | 0,08 | - |
| WY42 | S | S | S | S | S | S | S | S | S | S | S | R | E | 1 | 0,08 | - |
| WY43 | S | S | S | S | S | S | S | S | S | S | S | R | E | 1 | 0,08 | - |
| WY46 | S | S | R | S | S | S | S | S | R | S | S | R | E | 3 | 0,25 | + |
| WY47 | S | S | S | S | S | S | S | S | R | S | S | R | E | 2 | 0,17 | - |
| WY50 | S | S | R | S | S | S | S | S | R | R | S | R | E | 4 | 0,33 | + |
| AWY13 | S | R | R | S | S | S | S | S | S | R | R | R | E | 5 | 0,42 | + |
| AWY14 | S | S | S | S | S | S | S | S | S | R | S | R | E | 2 | 0,17 | - |
| AWY18 | S | S | S | S | S | S | S | S | S | R | S | R | E | 2 | 0,17 | - |
| AWY31 | S | S | S | S | S | S | S | S | S | R | R | R | E | 3 | 0,25 | + |

Footnotes: R- resistance, S – susceptible; CTX – cefotaxime, CAZ – ceftazidime, FEP – cefepime, AZT - aztreonam, ERT – ertapenem, IMP – imipenem, MEM - meropenem, CIP – ciprofloxacin, TET – tetracycline, C – chloramphenicol, CN – gentamicin, AK - amikacin, MDR – multidrug resistance strains
